# Genetic Linkage Mapping and Transmission Ratio Distortion in a Three-Generation Four-Founder Population of *Panicum virgatum* (L.)

**DOI:** 10.1534/g3.113.010165

**Published:** 2014-03-17

**Authors:** Guifen Li, Desalegn D. Serba, Malay C. Saha, Joseph H. Bouton, Christina L. Lanzatella, Christian M. Tobias

**Affiliations:** * Samuel Roberts Noble Foundation, Forage Improvement Division, Ardmore, Oklahoma 73401; † United States Department of Agriculture–Agricultural Research Service, Western Regional Research Center, Albany, California 94710

**Keywords:** biomass, feedstock, biofuel, allopolyploid

## Abstract

Switchgrass (*Panicum virgatum* L.), a warm season, C4, perennial grass, is one of the predominant grass species of the North American tall grass prairies. It is viewed as a high-potential bioenergy feedstock species because it can produce large amounts of lignocellulosic material with relatively few inputs. The objectives of this project were to develop an advanced switchgrass population and use it for the construction of genetic linkage maps and trait characterization. A three-generation, four-founder population was created and a total of 182 progeny of this advanced population were genotyped, including a mixture of self-pollinated and hybrid individuals. The female map integrated both subpopulations and covered 1629 cM of the switchgrass genome, with an average map length of 91 cM per linkage group. The male map of the hybrid progeny covered 1462 cM, with an average map length of 81 cM per linkage group. Average marker density of the female and male maps was 3.9 and 3.5 cM per marker interval, respectively. Based on the parental maps, the genome length of switchgrass was estimated to be 1776 cM and 1596 cM for the female map and male map, respectively. The proportion of the genome within 5 cM of a mapped locus was estimated to be 92% and 93% for the female map and male map, respectively. Thus, the linkage maps have covered most of the switchgrass genome. The assessment of marker transmission ratio distortion found that 26% of the genotyped markers were distorted from either 1:1 or 3:1 ratios expected for segregation of single dose markers in one or both parents, respectively. Several regions affected by transmission ratio distortion were found, with linkage groups *Ib-m* and *VIIIa-f* most affected.

Switchgrass (*Panicum virgatum* L.) is a morphologically diverse, warm season, C4, perennial grass and a predominant species of the tall grass prairies of North America (Bouton 2008). It has long been used for forage production and for soil conservation plantings due to its large fibrous root system, and it is now being developed as a bioenergy crop for second-generation biofuel production (Sanderson *et al.* 2006). Its suitability as a biomass feedstock is due to its high biomass yield potential, relatively simple growth requirements, and wide adaptation (Barney *et al.* 2009; Elbersen *et al.* 2001; Evanylo *et al.* 2005; Fike *et al.* 2006; Moser and Vogel, 1995). Efforts to improve yield, feedstock quality, and stress tolerance have recently been initiated and some of these efforts have focused on lowland ecotypes with high yield potential. These are tetraploid (2n = 4x = 36), whereas upland populations are both tetraploids and octoploids (2n = 8x = 72). Upland and lowland ecotypes can be distinguished genetically, based on morphology, and also by ecological preference (Casler 2005; Casler *et al.* 2004; Das *et al.* 2004). Lowland ecotypes are adapted to heavier soils, are tolerant of flooding, and are generally found in the warmer and wetter regions of the southern United States, whereas upland ecotypes are adapted to lighter soils and dry and cold zones of the middle and northern latitudes of the United States (Hopkins *et al.* 1996; Hultquist *et al.* 1996). Upland and lowland ecotypes occupy overlapping distributions, with significant gene flow between groups (Zhang *et al.* 2011).

To hasten efforts at efficient breeding, marker-based methods need to be adopted. Hence, the development of genomic resources and analysis of both structured and unstructured populations for genetic dissection of quantitative traits are priority research areas for switchgrass. One main goal of genetic mapping is to identify simply inherited markers that are closely linked to quantitative trait loci (QTL) (Jannink and Walsh 2002). A high-density genetic map of switchgrass is essential for effective application of marker-assisted selection (MAS) in breeding to increase the efficiency of selection and for characterization of specific regions in the genome that hold a special interest for map-based cloning. Mapping of molecular markers distributed throughout the genome of a plant species or at least around the gene of interest is a prelude for the application of molecular approaches in breeding switchgrass for improved yield and tolerance to various biological and environmental stresses.

The segregation patterns of markers in controlled biparental crosses of switchgrass are predicted to behave as a full-sib family derived from two heterozygous parents. The segregation may have different forms as the number of alleles or QTL segregating at a given locus may reach up to four and vary across loci in their informativeness (Wu *et al.* 2002, 2007). This introduces complexity to mapping and, in addition, linkage phase between QTL, and nearby markers may be unknown, which can introduce serious biases in estimations of QTL size and affect. A practical approach to this is to treat single-dose alleles segregating in each parent as dominant markers for the purposes of mapping in a two-way pseudo-testcross design (Grattapaglia and Sederoff 1994). Single-dose alleles in common to both parents, although less informative, can also be mapped with this approach. This is advantageous when there are insufficient numbers of fully informative markers (*ab* × *cd*) to integrate the parental maps. Transmission ratio distortion (TRD), deviation from the expected Mendelian ratio of segregating alleles at a locus (Sandler *et al.* 1959), may also affect estimates of recombination fraction. This phenomenon has been commonly encountered in mapping populations (Garcia-Dorado and Gallego 1992; Lorieux *et al.* 1995a,b) and is increasingly recognized as a potentially powerful evolutionary force that also affects the construction of genetic linkage maps (Lu *et al.* 2002).

Previous linkage mapping in switchgrass has been performed on a full-sib population of two lowland individuals (Okada *et al.* 2010), on a lowland × upland full-sib population (Missaoui *et al.* 2005; Serba *et al.* 2013), and on the selfed progeny of a heterozygous individual (Liu *et al.* 2012). Although self incompatibility systems are present in switchgrass (Martinez-Reyna and Vogel 2002), these incompatibility systems are not 100% effective. Up to 61% of self-fertilized individuals were reported in one controlled cross of a heterozygous northern lowland genotype, which allowed linkage map construction (Liu and Wu 2012; Liu *et al.* 2012). These studies have consistently demonstrated the nine base chromosomes of switchgrass are inherited in a disomic manner and have clearly demonstrated synteny with related grass species; however, there has not yet been definitive assignment of individual chromosomes to the two subgenomes. Here we describe joint linkage analysis in a cross that produced both self-pollinated and hybrid individuals from a population of four founders derived from the two linkage populations above with two parents and 182 progeny. The structure of this population enables marker phase to be unambiguously determined, particularly for those markers with alleles shared in common by both parents. The objectives of this project were to construct a genetic linkage map from this population with markers distributed throughout the switchgrass genome and to jointly map both self-pollinated and hybrid progeny as a prelude to analysis of loci underlying TRD and the analysis of QTL present in the parents.

## Materials and Methods

### Mapping population development

**Figure 1:**
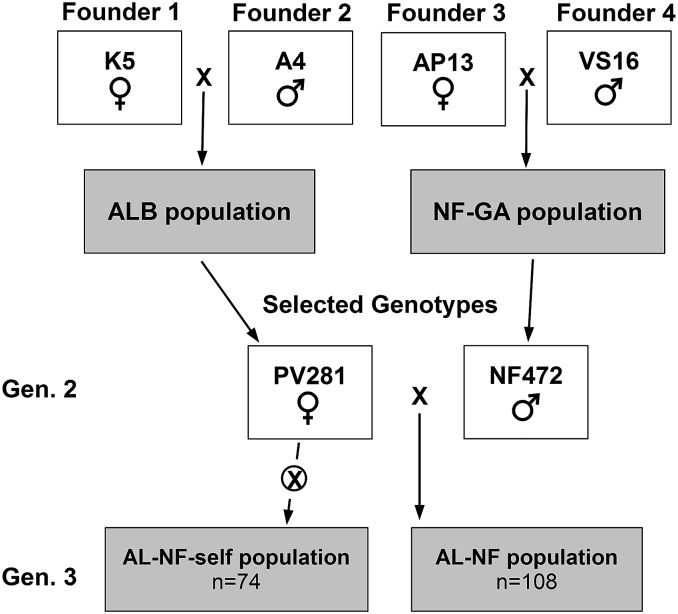
Switchgrass AL-NF mapping population development scheme using four founders in three generations. Other crosses were performed but the PV281 × NF472 cross resulted in sufficient seeds for further evaluation. AL-NF-self population represents progeny derived from self-pollination of PV281 that were initially identified by limited genotyping. AL-NF population represents hybrid individuals. The number (*n*) of individuals in each population is indicated.

This mapping population, designated AL-NF, was developed from four founders in three generations and, as such, is unique from previously reported mapping populations in switchgrass. First, selected genotypes from Kanlow (K5) and Alamo (A4) were crossed in Albany, California, to generate K5 (female) × A4 (male) (ALB) population (Okada *et al.* 2010). The F1 pseudo-testcross population was field evaluated in Athens, Georgia, and four distinct genotypes were selected. A second population (NF-UGA) was developed by crossing a lowland selection, AP13, (female) to an upland selection, VS16, (male), at the University of Georgia (Missaoui *et al.* 2005). The population was further expanded at the Noble Foundation and 251 F1 progenies have been field evaluated at three locations: Ardmore (34° 11′ 32′′ N and 97° 5′ 21′′ W) and Burneyville (33° 53′ 20′′ N and 97° 16′ 36′′ W), Oklahoma, and Watkinsville (33° 52′ 19′′ N and 83° 24′ 20′′ W), Georgia. Four distinct genotypes were selected. Genotypes selected from both populations were grown in greenhouses at the Noble Foundation and reciprocal crosses were made in the spring of 2008. A panicle from each of the female and male parents of the selected genotypes was put together in a paper bag. To enhance pollen shedding, the bags were manually tapped every morning. The tillers were tied to a supporting bamboo stick until seed maturity. The seeds were harvested from both the parents to obtain cross and reciprocal seed. The cross with the most seed set, *e.g.*, PV281 (female) (selection from ALB) × NFGA472 (male) (selection from NF-UGA), was selected to develop a three-generation and four-founder population (AL-NF). The crossing scheme for population development is illustrated in Figure 1.

Seeds collected from the cross were scarified in diluted sulphuric acid and chilled at 4° for 7 d. The seeds were germinated at 24° with a 16-hr photoperiod. Seedlings were grown in a greenhouse at 32° day and 21° night temperatures. The young plants were propagated by splitting the tillers and then set out in the field following an R-256 (251 progeny, two sets of parents and one Alamo check) honeycomb design. The population has been field evaluated for 3 yr at two locations in Oklahoma: Red River Farm (Burneyville) and Ardmore; 188 plants were randomly selected and genotyped for construction of genetic linkage maps.

### DNA extraction and quantification

Fresh tissue from young leaves of greenhouse-grown plants was collected and frozen in liquid nitrogen. The frozen samples were ground in 2-ml tubes with two zinc-plated ultra smooth ball bearings (Daisy Outdoor Products, Rogers, AZ) using a Tissuelyzer (Qiagen Inc., Valencia, CA). The genomic DNA was extracted using DNeasy Plant Mini Kit (Qiagen Inc.) following the manufacturer’s recommendation.

### Marker screening

Several sequence-based markers have been developed for switchgrass. In this study, 342 pairs of eSSR (expressed sequence tag–simple sequence repeat) primers developed from publicly available EST resources, 512 pairs of gSSR (genomic SSR) primers developed from (GA/CT)n enriched genomic libraries, and 144 pairs of STS primers developed from switchgrass cellulase and transferase gene sequences were screened for polymorphism. Primers were synthesized by Sigma-Aldrich Corporation (Woodlands, TX) with 18 nucleotides of M13 (5′-TGTAAAACGACGGCCAGT-3′) universal primer sequence added onto the 5′ end of the forward primer (Schuelke 2000). The M13 (-21) universal primer sequence labeled with PET, VIC, 6-FAM, or NED fluorescent dyes was synthesized by Sigma-Aldrich (St. Louis, MO). All of the 998 primer pairs were prescreened using a panel of two parents and six randomly selected F1 individual plants. The primers that were polymorphic in two parents and/or segregate in the progeny were used for genotyping the whole population.

### PCR protocol

Approximately 20 ng template genomic DNA was amplified in a total volume of 10 μl. PCR reactions consisted of 1× PCR buffer, 150 µM dNTPs, 0.25 µM forward primer and 1 µM reverse primer, 1 µM M13 florescent dye, and 0.5 U GoTaq DNA polymerase (Promega, Madison, WI) and brought to volume by adding ddH_2_O. The PCR were performed on a GeneAmp PCR System 9700 thermocycler (Life Technologies, Carlsbad, CA) following a touchdown PCR program. After an initial 3-min denaturation at 95°, the thermal profile consisted of three phases. The first 6 cycles consisted of a 45-sec denaturation step at 94° and 5-min annealing/extension at 68°, which was reduced by 2° per cycle. The next 8 cycles consisted of a 45-sec denaturation at 94°, followed by 2-min annealing step at 58° that was reduced by 1° per cycle, and extension step at 72° for 1 min. The final 25 cycles consisted of a 45-sec denaturation step at 94°, a 2-min annealing step at 50°, and a 1-min extension step at 72°. A final 7-min extension step at 72° was then performed. This method was observed to enrich the correct product over any nonspecific products amplified by the primers.

The amplification products were diluted in formamide and analyzed by capillary electrophoresis with labeled size standards using an ABI3730 and GeneMapper v.3.7 software (Life Technologies). All markers were scored as dominant loci. Markers with more than 15% missing data were removed from analysis.

### Identification of hybrid and self-fertilized populations

Switchgrass is highly cross-pollinated and known to be self-incompatible. However, varying degrees of self-fertilization have been observed (Okada *et al.* 2010; Liu and Wu 2012). To identify the selfed plants among the hybrids, the genomic DNA of the four founders, two parents, and 188 progeny were genotyped using 12 polymorphic SSR markers segregating in the male parent. The amplified product was visualized and scored as indicated above. The amplicons of each individual were compared with the two parents. Inbred individuals were identified as those that yielded female-specific but lacked male-specific amplicons.

### Linkage analysis

The linkage analysis was conducted using polymorphic amplicons referred to henceforth as “markers” that were single-dose (SD) in either parent and expected to segregate 1:1 or were shared by both parents (SD × SD) and expected to segregate 3:1. The SD × SD markers were placed on the map after the marker order created using only SD markers had been fixed. JoinMap 4.0 software (VanOoijen 2006) was used following our breeding full-sib family (CP) model or an F2 model for self-fertilized individuals using the regression mapping algorithm (Stam 1993). For the female map, mean recombination fractions and combined likelihoods of odds (LOD) values of markers from self and hybrid groups were used to integrate the separate linkage groups (LG) and produce the final maps. The SD markers were grouped into linkage groups at an independence test LOD threshold of 7.0. The calculations of the linkage map were performed using all pair-wise recombination frequency estimate of less than 0.40 and a LOD score more than 1.0 (ripple value = 1; goodness-of-fit jump threshold = 5; and a triple threshold = 5). The male and female hybrid maps were analyzed following the two-way pseudo-testcross strategy (Grattapaglia and Sederoff 1994). Ungrouped markers or misgrouped markers were assigned based on their strongest cross link (SCL). If the SCL was more than 7.0, then ungrouped or suspect markers were reassigned for both female and male segregating markers.

Genotyping data from six individuals were excluded from map construction due to either large amounts of missing data or inconsistent genotyping. Map construction for the NF472 map was conducted using marker data from 108 hybrid genotypes, whereas genotypic data from the entire population of 182 (74 self-fertilized and 108 hybrid) individuals were used for the PV281 map. The combined LOD values and mean recombination fractions were used to integrate the two maps using the regression mapping algorithm. The Kosambi mapping function (Kosambi 1944) was used to convert recombination units into genetic distances.

Switchgrass genome length was estimated by the formulae ∑*L*_i_[(*k*_i_+1)/(*k*_i_−1)] as proposed by Chakravarti *et al.* (1991) and *L* + (2*tL*)/*n* (Fishman *et al.* 2001), where *L* is the total length of the genetic map (cM), *n = k−t* is the number of marker intervals, *k* is the number of marker loci, *k*_i_ is the number of marker loci on the *i*th linkage group, where *i =* 1, 2,..., *t*, and *t* is the number of linkage groups. With the assumption of random distribution of marker loci in the genome, the proportion of the genome within *d* cM of a marker locus was estimated to be 1−*e*^-2^*^dk^*^/^*^G^*, where *k* is the number of mapped loci and *G* is the estimated genome length.

For determining homologies between linkage groups and deriving consistent nomenclature of subgenomes with previous linkage analysis (Okada *et al.* 2010), the populations were genotyped with subsets of previously mapped markers. Resolving homeologous relationships and alignment of parental LG was performed by the following: (1) alignment of multiple markers derived from the same primer set present on different LG; (2) assignment of LG based on identity with the founder’s linkage map order determined by Okada *et al.* (2010); and (3) placement of SD × SD markers. For those few LG with no markers in common, a sequence similarity-based strategy was used similar to one used with oat (Gutierrez-Gonzalez and Garvin 2011). Thus EST-STS markers were used to identify collinear stretches of foxtail millet (*Setaria italica*) genome as the criteria for homeologue formation. EST-STS marker sequences were compared using blastn to the foxtail millet genome (www.phytozome.net; Bennetzen *et al.* 2012) and 1.0×10^−5^ E-value threshold was used to determine synteny. When switchgrass marker sequences from ungrouped LG aligned to the same *S. italica* scaffold, irrespective of the position, these LG were considered homeologous/homologous. Following construction of the LG, map graphics were constructed using MapChart 2.2 (Voorrips 2002).

### Analysis of transmission ratio distortion

All markers were scored as dominant loci and those with TRD were identified on the basis of significant deviations from 1:1, 3:1, or 5:1 ratios expected based on allele dosage and disomic or tetrasomic inheritance using the χ^2^ test (*P* < 0.05). Markers displaying significant TRD were excluded from initial map construction and placed at their most likely position after the initial mapping step. To assess the extent of multilocus interactions between unlinked loci associated with single-locus segregation ratio distortion, all mapped TRD markers were tested for independence against all markers in other LG in both male and female maps using the two-locus genotypes in the two-by-two contingency χ^2^ test. Since comparisons were made among unlinked SD alleles, the significance was evaluated with a correction for 31,871 comparisons of 28 LG with at least one TRD marker with 35 other LG (*P* < 1.57 × 10^−6^). The distribution of the TRD markers was also assessed in the female and male maps, and when three or more TRD markers were clustered together we considered them a candidate TRD locus (Li *et al.* 2011).

### Comparative mapping

Individual maps across studies were compared based on their correlation coefficients for marker interval distance and for marker order based on their longest common subsequence (LCS) using the qualV package (Jachner *et al.* 2007). Results were expressed as markers present in the LCS as a fraction of the number of markers in common between groups. EST-SSR markers were compared to v. 2.1 of the *Setaria italica* genome (www.phytozome.net) using blast.

## Results

### Population development

Four F1 genotypes from each of ALB and NF-UGA populations were selected with distinct morphologies and a total of 10 successful crosses and reciprocal crosses were made between them. The highest number of seeds (850) was obtained from the cross PV281 × NFGA472 and the reciprocal cross NFGA472 × PV281 provided the second highest seed (746). Poor seed set was observed in the cross between the parents PV204 and NFGA472, where 25 seeds were obtained when NFGA472 served as the pollen donor and 33 were obtained when PV204 served as the pollen donor. The cross with the highest seed set (PV281 × NFGA472) was developed as the mapping population ALB × NF. In this cross, PV281 was the female parent and NFGA472 was the male parent.

### Markers and their inheritance

Among the 998 primer pairs, 447 were genotyped on the entire population. These consisted of 158 (46.2%) eSSR, 272 (53.1%) gSSR, and 17 (11.8%) STS primer pairs that produced a total of 1203 amplified size polymorphisms (referred to henceforth as markers) between the two parents (Table 1). The average numbers of polymorphic markers per primer set were 2.97, 2.03, and 1.97, respectively, for the gSSR, eSSR, and STS. Of the total markers, 484 segregated in the female (PV281) and 460 segregated in the male (NF472) parent. The remaining 259 markers were monomorphic in the parents but segregated in the progeny population (Table 2). This result indicated that there was high molecular marker polymorphism between the two parents.

**Table 1:** Molecular marker categories used, their amplification, and their polymorphism in the AL-NF mapping population

Marker Categories	Total Primers Screened	Polymorphic Primers	Average Polymorphic Markers	Polymorphism (%)
eSSR	342	158	2.03	46.2
gSSR	512	272	2.97	53.1
STS	144	17	1.94	11.8
**Total**	**998**	**447**	**2.69**	**44.8**

**Table 2:** Segregation of molecular markers genotyped in AL-NF and AL-NF-self populations

Marker	Female (PV281) Parent					Male (NF472) Parent					Both Parents				
Category	Loci	1:1	3:1	5:1	Distorted	Loci	1:1	3:1	5:1	Distorted	Loci	1:1	3:1	5:1	Distorted
eSSR	136	109	1 (1)*^a^*	4	22	130	78	8	3	41	106	2	55 (15)	9	39
gSSR	336	270	7 (5)	3	56	321	189	17 (4)	3	112	141	3	78 (25)	14	47
STS	12	11	0	0	1	9	7	1	0	0	12	0	5 (2)	3	4
Total	484	390	8 (6)	7	79	460	274	26 (4)	6	153	259	5	138 (42)	26	90

a Numbers in parentheses indicate number of markers fitting into both 3:1 and 5:1 segregation ratios.

In the hybrid population, single-dose markers in either parent are expected to have 1:1 segregation in the hybrid progeny, whereas double-dose markers in only one parent will be expected to segregate 3:1 under strict disomic inheritance or 5:1 under strict tetrasomic inheritance. The χ^2^ tests of markers segregating in the hybrid population indicated that 390 of the polymorphic markers present in the female parent and 274 markers in the male parent fit a segregation ratio of 1:1 in hybrid or 3:1 in self-pollinated progeny. In the hybrid population, there were 8 markers that fit a 3:1 ratio and 7 markers that fit a 5:1 ratio among those that segregated in the female parent; however, among those that segregated in the male parent there were 26 markers that fit a 3:1 ratio and 6 markers that fit a 5:1 ratio. The remaining 232 markers (79 in the female and 153 in the male parent) did not fit any expected segregation ratio. Among 259 markers monomorphic in the parents that segregated in the progeny, 138 fit a 3:1 ratio expected of a single dose marker heterozygous in both parents under either disomic or tetrasomic inheritance (Table 2). The remaining 90 markers did not fit any expected segregation ratio. In total, 26% of the markers in the hybrids were significantly distorted from expected segregation patterns.

In the AL-NF-self subpopulation, markers affected by TRD were not necessarily identical to those affected in the hybrid subpopulations. Overall, 39% of the mapped markers in this population did not fit an expected 3:1 ratio of these 200 markers; only 42 were also affected by TRD in the hybrid subpopulation (data not shown).

### Construction of parental linkage maps

**Figure 2:**
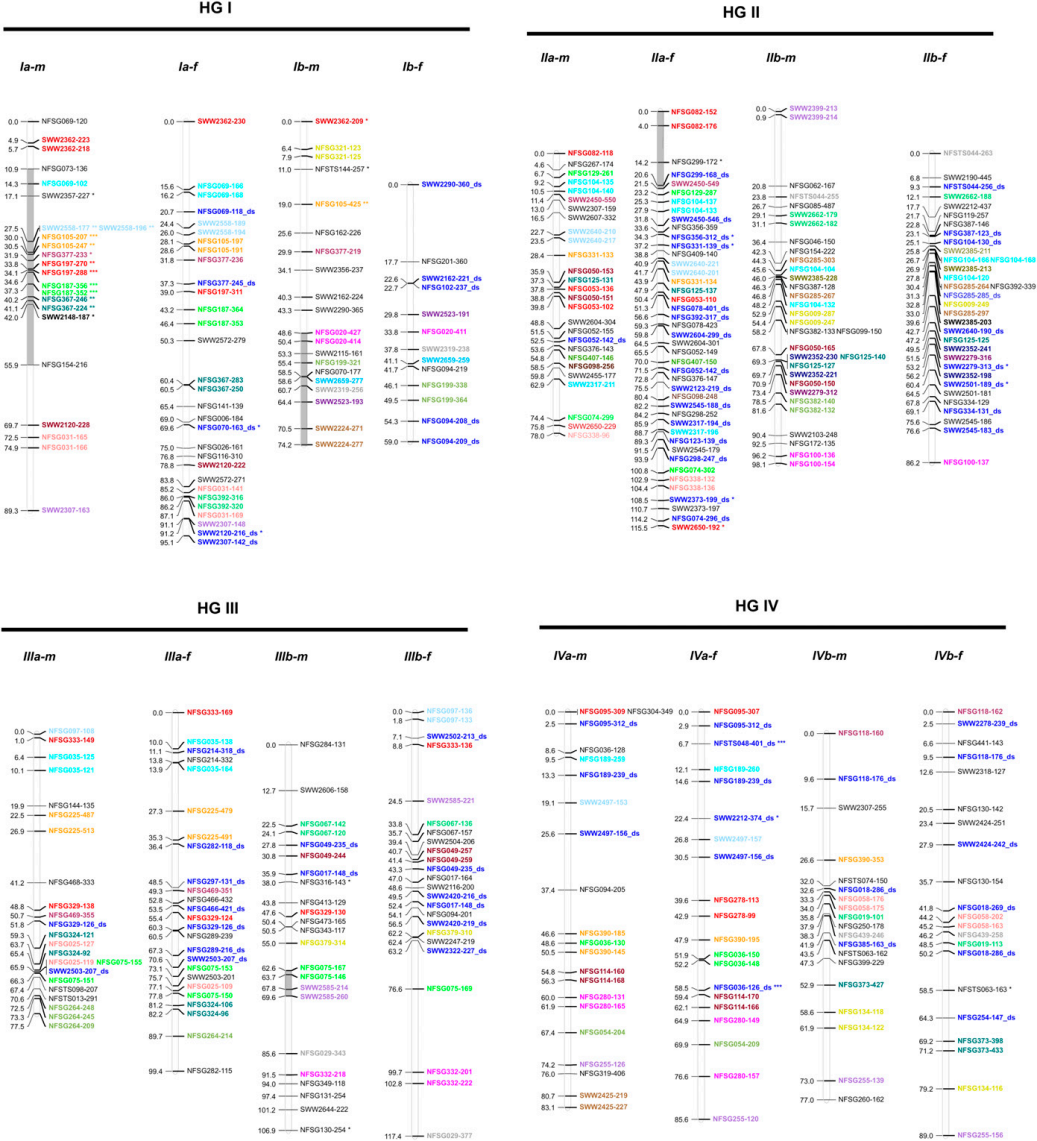

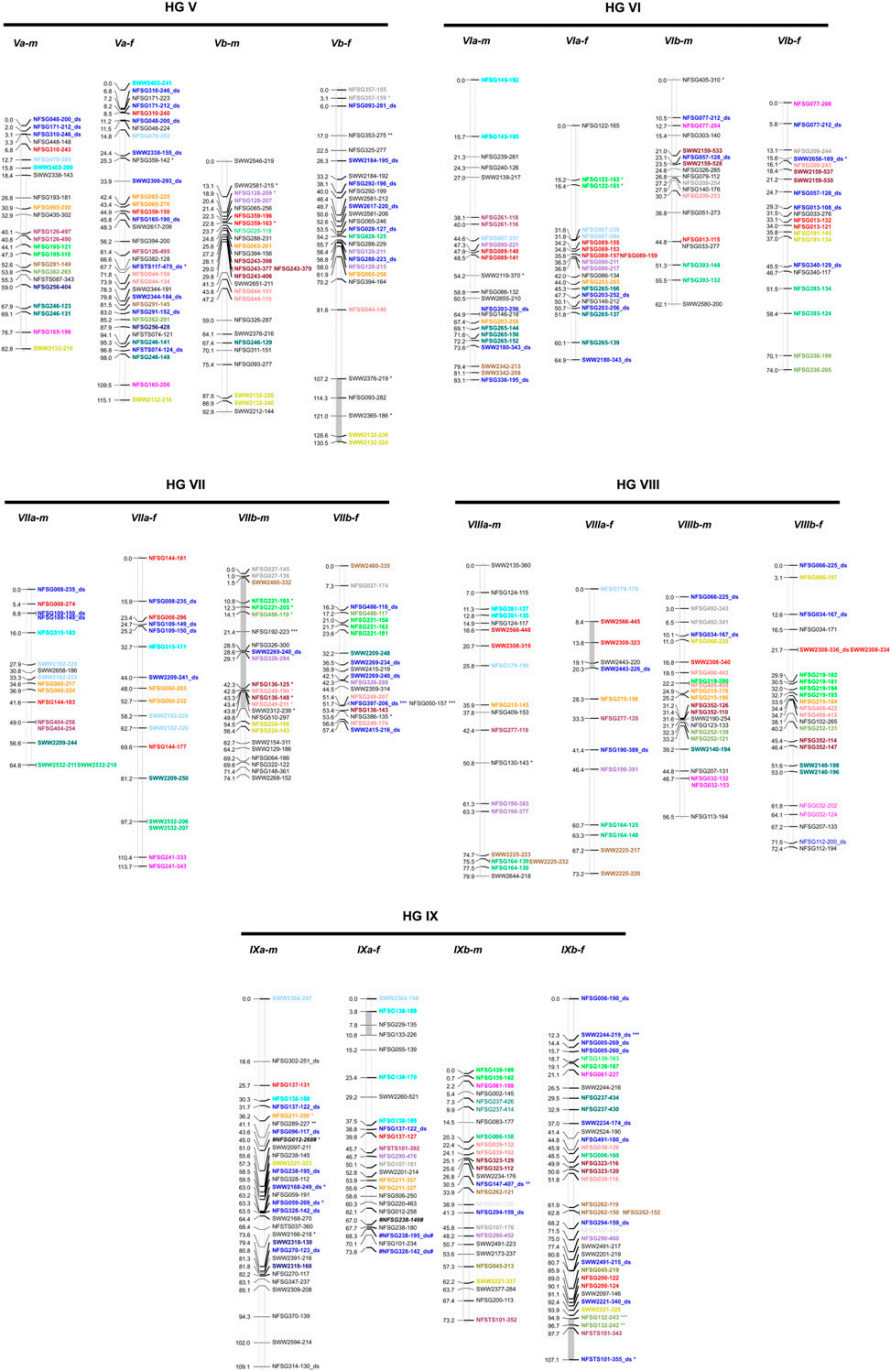
Consensus linkage map of PV281 and NF472. PV281 linkage groups were constructed by integrating genotypic data from both self-fertilized individuals (*n* = 74) and PV281 × NF472 hybrid individuals (*n* = 108) (see *Materials and Methods*). The Roman numeral designation of each homology group (I–IX) follows the foxtail millet chromosomes. NF472 linkage groups were constructed based only on hybrid individuals as it served as the male donor in the cross. Individual linkage groups are indicated by vertical bars. Numbers to the left of the bar indicate position in cM, whereas marker names are indicated to the right of the bar. Markers ending in “ds” are double simplex markers present in both parents. Markers used to align the subgenomes were amplicons of different fragment length detected with the same primer set. These markers are set-off by similar color labeling. The letters (*f*) and (*m*) at the end of the linkage group name denote female (PV281) and male (NF472) parental maps, respectively. Severely distorted markers are noted with asterisks: *P = 0.01, **P = 0.001, and ***P = 0.0001. Shaded regions of the individual linkage groups indicate the presence of three or more consecutive markers with TRD. Markers with significant interactions on different linkage groups are indicated by a hash symbol (#).

The molecular markers used to produce the parental linkage maps and the genome coverage are summarized in Table 3. The linkage analysis of 527 polymorphic markers for the female parent map produced from both AL-NF and AL-NF-self subpopulations consisted of 375 SD markers and 111 SD × SD markers that included data from 255 different primer pairs. These were formed into 18 linkage groups. Among the 445 mapped markers in the female map, 318, 116, and 10 were gSSR, eSSR, and STS markers, respectively. A total of 82 SD markers remained ungrouped. The female map spanned 1629 cM. Average length of each LG was 91 cM. An average of 25 polymorphic markers per LG with an average density of 3.8 cM per marker was mapped. The range of polymorphic markers mapped was from 13 to 43 per LG. The longest linkage group was *Vb-f*, with a total length of 131 cM, whereas the shortest was *IVa-f*, which had a total length of 65.6 cM (Table 4 and Figure 2).

**Table 3:** Summary of parental genetic linkage maps of the AL-NF-self and hybrid populations

Mapping Features	Female (PV281) Map	Male (NF472) Map
Total number of linkage groups	18	18
Total map length, cM	1629	1462
Average linkage group length, cM	91	81
Total number of loci mapped	445	417
Average loci per LG, n	25	23
SD markers mapped	293	315
SD × SD mapped	111	36
Unmapped SD markers	82	51
Estimated genome length, cM	1766	1543
Percent genome coverage within 5 cM mapped loci	91.8	92.7

**Table 4:** Number of markers, linkage group length, and marker density comparisons between female (PV281) and male (NF472) parental linkage groups

PV281				NF472			
LG	Mapped Markers	Total Length (cM)	Average Interlocus Distance	LG	Mapped Polymorphic Markers	Total Length (cM)	Average Interlocus Distance
*Ia-f*	30	95	3.2	*Ia-m*	23	89	3.9
*Ib-f*	13	59	4.5	*Ib-m*	20	74	3.7
*IIa-f*	43	115	2.7	*IIa-m*	27	78	2.9
*IIb-f*	33	86	2.6	*IIb-m*	32	98	3.1
*IIIa-f*	25	99	4.0	*IIIa-m*	23	78	3.4
*IIIb-f*	24	117	4.9	*IIIb-m*	23	107	4.6
*IVa-f*	20	86	4.3	*IVa-m*	21	83	4.0
*IVb-f*	21	89	4.2	*IVb-m*	19	77	4.1
*IXa-f*	24	74	3.1	*IXa-m*	31	109	3.5
*IXb-f*	37	107	2.9	*IXb-m*	26	73	2.8
*Va-f*	34	115	3.4	*Va-m*	23	83	3.6
*Vb-f*	27	131	4.8	*Vb-m*	26	93	3.6
*VIa-f*	20	65	3.2	*VIa-m*	24	83	3.5
*VIb-f*	20	74	3.7	*VIb-m*	18	62	3.4
*VIIa-f*	17	114	6.7	*VIIa-m*	16	65	4.1
*VIIb-f*	20	57	2.9	*VIIb-m*	24	74	3.1
*VIIIa-f*	13	73	5.6	*VIIIa-m*	19	80	4.2
*VIIIb-f*	24	72	3.0	*VIIIb-m*	22	57	2.6
Average	25	91	3.8		23	81	3.7
Total	445	1629			417	1462	

Similarly, linkage analysis of 453 polymorphic markers segregating in the male parent map allowed mapping of 315 SD markers and 36 SD × SD markers, and the resulting 18 linkage groups included data from 284 different primer pairs. The numbers of gSSR, eSSR, and STS markers were 306, 102, and 9, respectively. The male map spanned a total length of 1462 cM, with the average length of 81 cM and an average of 23 polymorphic markers per LG. The number of polymorphic markers mapped per LG ranged from 16 to 32, with LG lengths ranging from 57 cM (*VIIIb-m*) to 109 cM (*IXa-m*). Marker density ranged from 2.6 to 4.2, with an average of 1.46 mapped markers per primer pair.

The distribution of the three types of markers among the linkage groups was random and no aggregation of genomic or EST markers was observed. The STS markers were mapped in seven LG of both the female and male maps. In the female map, STS markers were placed in LG *IIb-f*, *IVa-f*, *IVb-f*, *Va-f*, *IXa-f*, and *IXb-f*, whereas in the male map they were placed in LG *Ib-m*, *IIb-m*, *IIIb-m*, *IVb-m*, *Va-m*, *IXa-m*, and *IXb-m*.

### Genome length and coverage

As estimated from the information of the parental maps, total genome lengths of switchgrass were 1776 cM and 1596 cM for the female map and male map, respectively. Two methods of estimation were used and consistent genome length estimates were obtained using both Fishman *et al.* (2001) and Chakravarti *et al.* (1991) methods. The proportion of the genome within 5 cM of a marker locus was estimated to be 92% for the female map and 93% for the male map.

The average of two genome length estimates (1686 cM) was taken as the expected genome length of switchgrass. Based on an estimated genome size of ∼1600 Mbp for tetraploid switchgrass (Saski *et al.* 2011), the average recombination rate across all LG was approximated at 1.05 cM per Mbp.

### Map integration

The linkage groups were integrated with one another based on shared SD × SD markers, or SD markers co-amplified by the same primer set that represented either homeologous loci or alternate alleles heterozygous in either both subgenomes or both parents, respectively (Figure 2). Accordingly, nine homology groups of four linkage groups each were formed. All groups were then assigned names consistent with previous nomenclature based on marker alleles shared in common with the linkage map of Okada *et al.* (2010) and synteny of selected eSSR markers sequences with the foxtail millet (*Setaria italica*) pseudomolecules (http://www.phytozome.net v7.0). The nine homology groups formed represent the base chromosome number for switchgrass.

### Transmission ratio distortion

We found that 26% of the genotyped markers were affected by TRD; however, only 17% of the markers included in the final maps were distorted. The rest could not be positioned with certainty. TRD markers that affected the AL-NF (PV281 × NF472) map were compared with the two related switchgrass pseudo-testcross populations that it was developed from: ALB (A4 × K5) and NF-UGA (AP13 × VS16). As compared to the ALB population in which 8.7% of the markers were affected by TRD, the AL-NF and NF-UGA populations showed a higher frequency of TRD markers (Table 5), with the highest rates (25.1%) found in the NF-UGA population. A total of 143 TRD markers were recorded in the AL-NF population. Of these, 69 segregated in the female and 70 segregated in the male parent, whereas 4 were distorted SD × SD markers that segregated in both parents. In the male map 44% of the SD TRD markers were scored as present in excess, whereas in the female map the percentage of SD TRD markers present in excess was 54%.

**Table 5:** Number and frequency of molecular markers with transmission ratio distortion in three switchgrass pseudo-testcross mapping populations

Population	Type	Total	Distorted (%)
ALB	gSSR	509	40 (7.86)
	STS	55	4 (7.3)
	eSSR	945	88 (9.31)
Total		1509	132 (8.7)
NF	gSSR	874	222 (25.4)
	STS	36	11 (30.6)
	eSSR	168	38 (22.6)
Total		1078	271 (25.1)
AL-NF	gSSR	602	94 (15.6)
	STS	19	6 (31.6)
	eSSR	214	43 (20.1)
Total		835	143 (17.1)

Distorter regions consisting of clusters of three or more consecutive TRD markers were observed on LG *IIa-f*, *Vb-f*, *VIIIa-f*, *IXa-f*, *IXb-f*, and *VIIIa-f* in the PV281 map, indicating a biological phenomenon as opposed to genotyping error. Similarly, in the NF472 map LG *Ia-m*, *Ib-m*, *IIIb-m*, *and VIIb-m* were affected by distorter regions that are indicated in Figure 2. In three cases, genotypic information from the founders in these distorter regions allowed determination of which haplotype was present in excess. Regions affected by TRD in LG *VIIb-m* and *IIIb-m* were skewed toward the AP13 haplotype, whereas the TRD region on *Ia-m* was skewed toward the VS16 haplotype. Significant interactions between loci on corresponding LG in the male and female maps in groups *IXa-m/f* and *VIa-m/f* between NFSG012-268 on LG *IXa-m* and the three adjacent markers NFSG238-195_ds, NFSG238-146, and NFSG328-142_ds on LG *IXa-f* as well as between NFSG007-357 on LG *VIa-m* and NFSG203-256_ds on LG *VIa-f* were observed. These interactions are indicated in Figure 2. There were no significant interactions between TRD markers and markers on different homology groups. Comparison of TRD with the related F1 population of Okada *et al.* (2010) demonstrated that LG *Ia-m* and *Ib-m* were affected by distortion in both maps, as was LG *VIIb-m*.

### Map comparisons

eSSR, gSSR, and STS primer pairs used in several other switchgrass linkage studies with the ALB population (Okada *et al.* 2010), the NF population (Serba *et al.* 2013), and the unrelated NL94 population (Liu *et al.* 2012) were compared using a string order comparison based on the LCS (Agarwala *et al.* 2000). This method identified the largest number of sequences or primer sets that had the same relative order between two different linkage groups. The LCS numbers were summed across all LG by subgenome and male or female linkage maps where appropriate and then expressed as a fraction of the total number of SSR shared in common. These results are presented in Table 6. In 75% of the cases it was not possible to compare *a* and *b* sub-genomes due to too few SSR shared in common. The percent of SSR in the LCS ranged from 47% to 77%, whereas the number of SSR mapped in common ranged from 3 to 103. Within the AL-NF population the LCS ranged between 0.74 and 0.57, with a maximum of 159 and a minimum of 24 SSR shared in common between subgenomes and male or female linkage maps. The correlation coefficient between AL-NF marker distances intervals was 0.77 for the NF population and 0.40 for the NF94 population.

**Table 6:** LCS map comparisons across all linkage groups*^a^*

	AL-NF*a-f*	AL-NF*a-m*	AL-NF*b-f*	AL-NFb-m
ALB*a-f* *^b^*	0.68 (98)*^c^*	0.72 (86)	— (17)	0.59 (27)
ALB*a-m*	0.77 (96)	0.68 (89)	0.69 (23)	0.68 (25)
ALB*b-f*	— (17)	0.53 (32)	0.58 (103)	0.58 (100)
ALB*b-m*	— (15)	— (16)	0.67 (94)	0.67 (90)
NF*a-f*	0.53 (99)	0.60 (88)	— (10)	— (13)
NF*a-m*	0.57 (75)	0.6 (55)	— (13)	— (10)
NF*b-f*	— (11)	— (7)	0.49 (81)	0.60 (84)
NF*b-m*	— (18)	0.71 (21)	0.48 (77)	0.59 (83)
NL94*a*	0.52 (36)	0.56 (39)	— (3)	— (5)
NL94*b*	— (16)	— (13)	0.47 (46)	0.6 (35)
*S. italica*	0.88 (18)	0.84 (19)	0.76 (21)	0.91 (23)

a Linkage mapping studies on populations ALB (Okada *et al.* 2010), NF (Serba *et al.* 2013), and NL94 (Liu *et al.* 2012) were compared with the AL-NF population using the LCS method and SSR primer sets used in multiple studies. The *Setaria italica* genome was compared to the AL-NF population using the molecular coordinates on the scaffold sequences of the best blast hits to EST-SSR.

b Italicized letters *a* and *b* refer to individual subgenomes, whereas *m* and *f* refer to male and female linkage maps.

c The first number represents the fraction of SSR compared that are present in the LCS, whereas the number in parentheses is the number of SSR shared in common between the two groups. LCS of groups with 18 or fewer markers in common was not reported.

Similar comparisons to the foxtail millet genome sequence were performed based on the top scoring blast hits of EST-SSR sequences to the foxtail millet genome sequence. The *e*-values of these results were less than 1 × 10^−40^ except for sww2209, which was 2 × 10^−12^. Comparisons were limited to EST-SSR markers and thus there were fewer markers present in the LCS, which contained between 76% and 91% of the total number (Table 6).

## Discussion

### Population development

The mapping population was developed in three generations using four switchgrass genotypes selected from four founders. Two of the genotypes, A4 and AP13, were selected from Alamo. The other genotypes, K5 and VS16, were selected from Kanlow and Summer, respectively. Alamo and Kanlow are lowland cultivars that have robust growth and high biomass yield potential, whereas Summer is a northern-adapted upland cultivar with limited biomass yield but cold tolerance.

These populations combine the variability from different founder populations and will be useful for detecting QTL for yield and other relevant traits. Knowledge of the linkage phase is critical for QTL mapping and other applications. In an outcrossing pedigree, the linkage phase of the parents is not known. Genotyping the grandparents allows determination of the linkage phase of the parents using identity by descent and yields improved results where there is limited marker coverage over inferring phase based on independence LOD scores of marker pairs.

### Selfed individuals

The presence of 74 self-pollinated individuals among the genotyped population requires a slightly modified mapping approach but also provides an opportunity to compare traits potentially impacted by inbreeding depression in related populations with different degrees of heterozygosity. The true rate of outcrossing in this population was not directly determined because differences in fitness at early life stages biased establishment and genotyping of the population. However, those individuals that were genotyped consisted of 59% hybrid and 41% selfed individuals. This indicates that the female parent of the cross (PV281) was effectively self-compatible. There have been several studies examining reproductive systems in switchgrass. These have found that both pre- and post-zygotic barriers to selfing are active, and that bagged inflorescences produce far fewer seeds than open pollinated inflorescences (Martinez-Reyna and Vogel 2002; Talbert *et al.* 1983). Estimates of 0.35% self-compatibility were obtained from 17 upland and lowland tetraploid genotypes by Martinez-Reyna and Vogel (2002).

The discoveries of conditionally compatible genotypes NL94 LYE 16×13 and SL93 7×15 that are self-compatible under controlled conditions but effectively self-incompatible in the field (Liu *et al.* 2013) as well as of the high self-compatibility found in PV281 demonstrate the existence of both reproductive modes in switchgrass. However, very little information is available regarding the genetic basis of incompatibility in switchgrass or other polyploid species. Loss of self-incompatibility due to modifiers of major SI loci, nonfunctional incompatibility alleles, or decreased competition from unrelated pollen at the stigma surface or stylar transmitting tissue under controlled conditions may play roles in conditional self-fertility in switchgrass.

### Marker diversity and segregation

The marker systems utilized in this study were SSR and STS markers. The SSR markers were developed from both genomic and EST sequences. The gSSR markers were developed from (GA/CT)_n_ enriched libraries. The eSSR markers were developed from publicly available switchgrass EST sequences. SSR markers have become quite useful in various aspects of molecular genetic studies, including assessment of genetic diversity (Amsellem *et al.* 2001; Ashley *et al.* 2003), ecological–genetic studies (Muraya *et al.* 2011), marker-assisted selection (Fazio *et al.* 2003), and genetic linkage mapping (Akkaya *et al.* 1995; Broun and Tanksley 1996). SSR have an additional evolutionary role in creating and maintaining quantitative genetic variation (Kashi *et al.* 1997).

Both gSSR and eSSR markers were used in the construction of this linkage map to cover genic as well as the intergenic regions of switchgrass. The gSSR markers proved to be evenly distributed throughout the genome. Sequence-tagged markers such as eSSR and STS markers have special importance in covering the euchromatic regions of genome (Mun *et al.* 2006; Ren *et al.* 2009) and reveal variation in transcribed genes among individuals (Ramchiary *et al.* 2011). Totals of 136 and 130 eSSR markers were placed on the female map and male map, respectively. In addition, 12 and 9 STS markers were also mapped on the female map and male map, respectively. These STS markers were developed from cellulase and transferase gene sequences and have special importance in mapping the genome region and genes involved in cellulose accumulation in switchgrass. It will be interesting to see co-segregation of any of these markers with QTL associated with biomass quality traits.

Switchgrass is an outcrossing plant; therefore, the parental genotypes are heterozygous. In a cross between such heterozygous parents, many single-dose polymorphic markers will amplify in one parent but will be absent in the other; therefore, they are expected to segregate in a 1:l ratio in their progeny (Grattapaglia and Sederoff 1994). Other markers are heterozygous in both the parents and expected to segregate 3:1 under disomic inheritance or 5:1 under tetrasomic inheritance models. The majority of the SSR and STS markers assayed in this population were found to follow the expected 1:1 ratio for the SD markers and 3:1 ratio for the SD × SD cross. This agrees with previous findings that switchgrass inheritance is disomic.

### Linkage map and genome coverage

The maps lengths produced by this study were similar to those reported in the NF population in which the female linkage map was 13% larger than the male map. In this study, the female linkage map was 11% larger than the male linkage map. Similarly, the overall map lengths were comparable, with this genetic linkage map being 90% of the length of that reported by Serba *et al.* (2013). These map lengths were both significantly smaller than that reported for the NL94 population. The relative lengths of the male and female linkage maps in this study were the opposite of the ALB population in which the male map was found to be relatively shorter. These results suggest that meiosis in the NF and AL-NF populations was behaving in a consistent manner, and that inclusion of increasing percentages of TRD markers in the individual switchgrass linkage studies did not appear to greatly inflate map length, consistent with the theory of Hackett and Broadfoot (2003).

### Transmission ratio distortion

Distorter loci affecting the transmission of closely linked markers are commonly present in mapping populations and can be linked to pre- or post-zygotic mechanisms, including pollen tube competition (Mangelsdorf and Jones 1926), pollen lethals (Rick 1966), preferential fertilization (Gadish and Zamir 1987), and selective elimination of zygotes (Rick 1963). Selfish genetic elements are also selected for in the asymmetric female meiosis of most plants and animals as meiotic drive. In the AL-NF population, TRD reached 17.1% in mapped markers. These rates of distortion are similar to that observed in the NF-GA mapping population (Serba *et al.* 2013). However, Okada *et al.* (2010) observed an SD of only 9% for the single dose markers of genomic and eSSR markers. This lower SD in the ALB population is attributed to the omission of highly distorted markers from the attempted mapping rather than significant biological differences. The distorted markers were distributed throughout the maps. There were several clusters of three or more loci in the female map and male map. In the female map TRD regions were observed on six LG, whereas TRD regions were observed on 11 LG in the male map. Significant interactions between markers in male and female LG *VI* and *IX* in the AL-NF population indicate possible post-zygotic interactions. The presence of TRD loci may lead to bias in estimation of the recombination fraction when two or more TRD loci are present on a single chromosome (Lorieux *et al.* 1995a,b). However, in our study we did not detect this situation, leading us to believe that our maps were not biased due to TRD.

## Conclusions

A three-generation and four-founder mapping population was developed in tetraploid switchgrass. We have constructed genetic linkage map using a combination of SSR and STS markers, with marker density close to saturation. The LG were arranged in nine homeologs corresponding to the haploid chromosome number of tetraploid switchgrass. Clustering of TRD markers was observed in some genomic regions. Using parental maps to identify QTL underlying agronomic traits for MAS is in progress. Due to the long establishment time of switchgrass and the need for multiple-year yield data, this approach has high potential to speed selection.
